# Insights into antioxidant activities and anti-skin-aging potential of callus extract from *Centella asiatica* (L.)

**DOI:** 10.1038/s41598-021-92958-7

**Published:** 2021-06-29

**Authors:** Visarut Buranasudja, Dolly Rani, Ashwini Malla, Khwanlada Kobtrakul, Sornkanok Vimolmangkang

**Affiliations:** 1 Department of Pharmacology and Physiology, Faculty of Pharmaceutical Sciences, Chulalongkorn University, Bangkok, 10330 Thailand; 2 Department of Pharmacognosy and Pharmaceutical Botany, Faculty of Pharmaceutical Sciences, Chulalongkorn University, 254 Phayathai Road, Patumwan, Bangkok, 10330 Thailand; 3 Research Unit for Plant-Produced Pharmaceuticals, Faculty of Pharmaceutical Sciences, Chulalongkorn University, Bangkok, 10330 Thailand; 4 Graduate Program in Pharmaceutical Science and Technology, Faculty of Pharmaceutical Sciences, Chulalongkorn University, Bangkok, 10330 Thailand; 5 Research Unit for Natural Product Biotechnology, Faculty of Pharmaceutical Sciences, Chulalongkorn University, Bangkok, 10330 Thailand

**Keywords:** Plant biotechnology, Preventive medicine, Plant biotechnology, Preclinical research, Drug development, Pharmacology

## Abstract

Formation of oxidative stress in dermal fibroblasts plays crucial roles in aging processes of skin. The use of phytochemicals that can promote capacity of fibroblasts to combat oxidative stress is an attractive strategy to prevent skin aging and promote skin beauty. *Centella asiatica* has been used to treat multitude of diseases for centuries. Previous investigations demonstrated that extracts from *C. asiatica* have a broad range of beneficial activities through their antioxidant activity. Hence, the extract from this medicinal plant could be a great candidate for anti-skin-aging agent. Callus culture offers a powerful platform for sustainable, rapid and large-scale production of phytochemicals to serve extensive demands of pharmaceutical and cosmeceutical industries. Here, we demonstrated the application of callus culture of *Centella asiatica* to produce bioactive metabolites. The 50% ethanolic extract of callus culture has distinctive features of chemical compositions and biological profiles. Information from HPTLC-DPPH and HPLC analysis suggested that the callus extract comprises distinctive antioxidant compounds, compared with those isolated from authentic plant. Moreover, results from cell culture experiment demonstrated that callus extract possesses promising antioxidant and anti-skin-aging activities. Pre-treatment with callus extract attenuated H_2_O_2_-induced-cytotoxicity on human dermal fibroblasts. The results from RT-qPCR clearly suggested that the upregulation of cellular antioxidant enzymes appeared to be major contributor for the protective effects of callus extract against oxidative stress. Moreover, supplementation with callus extract inhibited induction of matrix metalloprotease-9 following H_2_O_2_ exposure, suggesting its potential anti-skin-aging activity. Our results demonstrate the potential utility of *C. asiatica* callus extract as anti-skin-aging agent in cosmeceutical preparations.

## Introduction

Oxidative stress is a cellular phenomenon caused by a redox imbalance between accumulation of reactive oxygen species (ROS) and capacity of cells to detoxify these harmful agents. The excessive induction of oxidative stress is one of the major driving forces of several pathological condition, including aging processes of skin^1^. Fibroblasts in dermal layer of human skin is the principal target of oxidative stress^2^. Induction of oxidative stress in fibroblasts following exposure to external inducers e.g. ultraviolet radiation^3^, tobacco smoke^4^, and air pollutants^5^, causes a reduction in biosynthesis of collagen as well as a promotion of degradation of collagen. This aberrant homeostasis of collagen weakens structural integrity and mechanical properties of dermal skin, which eventually resulting in the emergence of aging characteristics, e.g. formation of wrinkle and loss of skin elasticity^6^. Hence, the use of compounds or extracts that can enhance the capacity of fibroblasts to neutralize the oxidative stress would be an attractive approach for preventing age-related skin disorders.

*Centella asiatica *(*L.*) is a medicinal herb with high economic value. This plant has been traditionally used for treatment of variety diseases in Chinese and Ayurvedic medicines for centuries^7^. The major bioactive metabolites for *C. asiatica* are centelloids, including asiaticoside, madecassoside, asiatic acid and madecassic acid. These centelloids have been widely used as important biomarker for *C. asiatica*. Extracts from *C. asiatica* demonstrates a broad range of pharmacological effects through their antioxidant activities in several preclinical models. Treatment with water extract of *C. asiatica* prevented mitochondrial dysfunctions of isolated neurons through an attenuation of oxidative stress^8^. Ethanolic extract of *C. asiatica* decreased oxidative stress *via* an enhanced activities of cellular antioxidant enzymes in Parkinson’s model of mice^9^. Oral administration of water extract of *C. asiatica* suppressed lipid peroxidation in an *in vivo* model of hyperlipidemia^10^. Supplementation of standardized extract of *C. asiatica* inhibited rotenone-induced-hepatotoxicity by inhibition of lipid peroxidation *in vivo*^11^. Altogether, extract from *C. asiatica* has a potential therapeutic utility on oxidative stress-related-disorders, including skin aging.

Naturally grown plant serves as source for secondary metabolites which can be used in cosmeceutical and pharmaceutical industries. However, the major limitations for production of compounds from wild natural plant are the dependence of geographical, seasonal and environmental factors. In addition, further drawbacks for extraction and purification of secondary metabolites from intact plants include complex and time-consuming process; and low concentration of bioactive compounds^12^. To overcome these obstacles, *in vitro* callus culture has been proposed to be an alternative procedure to efficiently produce bioactive metabolites. This *in vitro* method offers a manufacturing system which ensures the continuous supply of compounds with uniform quality and high yields. With this approach, cells of any plants, even rare or endangered species, can be easily manipulated and maintained to produce compounds of interest. The active compounds can be produced independently of external factors. Moreover, plant cell culture uses aseptic technique so it will not be threatened by micro-organisms or pests^13^. The maintenance of aseptic conditions is essential for successful tissue culture procedures^14^. Taken together, plant cell culture represents an attractive platform for mass production of phytochemicals.

In this present study, we demonstrated the application of callus culture of *C. asiatica* to produce bioactive metabolites. The major goals of this study are (1) to evaluate the antioxidant capacities of callus extracts; (2) to investigate the beneficial activities of extracts against oxidative stress in human dermal fibroblasts. Data from our study will provide an invaluable insight into potential application of *C. asiatica* callus extract in anti-aging cosmeceutical products.

## Material and methods

### Chemical and reagents

Asiaticoside (98%; Cat no. 43191), asiatic acid (97%; Cat no. A2612), madecassoside (95%; Cat no. M6949), madecassic acid (90%; Cat no. PHL80229), kaempferol (97 %; Cat no. 60010), quercetin (95%; Cat no. Q4951), rutin (95%; Cat no. R5143), polyethylene glycol (PEG; Cat no. 95904), 2-aminoethyl diphenylborinate (Cat no. D9754), sucrose (Cat no. 1076535000) hydrogen peroxide (H_2_O_2_; Cat no. 1072090500), 3-(4,5-dimethylthiazol-2-yl)-2,5-diphenyltetrazolium bromide (MTT; Cat no. M2128), 2,2-diphenyl-1-picrylhydrazyl (DPPH; Cat no. 300267), adenosine 5′-triphosphate (ATP) disodium salt hydrate (Cat no. A2383), *p*-anisaldehyde (Cat no. A88107), dimethyl sulfoxide (DMSO; Cat no. 102952), dichloromethane (Cat no. DX0835), ethyl acetate (Cat no. 107048), and HPTLC silica gel 60 F_254_ (Cat no. 105642) were purchased from MilliporeSigma (Burlington, MA, USA). HPLC-grade acetonitrile (Cat no. 10754361), ethanol (Cat no. 10538071), acetic acid (Cat no. 14650388) and methanol (Cat no. 10675112) were obtained from J.T. baker (Phillipsburg, NJ, USA). Cell culture media and supplements (minimum essential medium (MEM), fetal bovine serum (FBS), pyruvate, and penicillin/streptomycin) were purchased from Thermo Fisher Scientific (Waltham, MA, USA). All other chemicals in this study were analytical grade. Murashige and Skoog medium (MS; Cat no. M519), 1-naphthaleneacetic acid (Cat no. N605), 6-benzylaminopurine (Cat no. B800) and Agargellan^TM^ (Cat no. A133) were obtained from Phytotechnology Labs (Lenexa, KS, USA).

### Plant material

The explants were obtained from in vitro germinated *C. asiatica* seedlings. *C. asiatica* is a commercially available plant and it is grown at the garden of Faculty of Pharmaceutical Sciences, Chulalongkorn University, Thailand (Latitude-13.7440° N and longitude- 100.5307° E). Experimental research on plants complies with relevant institutional, national, and international guidelines and legislation. The seed of the plant used in the study was collected under the permission of the Department of Pharmacognosy and Pharmaceutical Botany, Faculty of Pharmaceutical Sciences, Chulalongkorn University, Thailand. The plant was identical to a voucher specimen (herbarium number 010018) deposited at Museum of Natural Medicines, Chulalongkorn University, Thailand. To remove dust particles, collected seeds were washed with detergent for 10 min. The pre-washed seeds were sterilized with 70% ethanol for 1 min, followed by 20% commercial bleach solution for 10 min. All subsequent steps were performed under a laminar flow cabinet. The seeds were washed five times with sterile distilled water to remove all traces of sterilant. The sterilized seeds were then inoculated on Murashige and Skoog medium, supplemented with 0.5% Agargellan^TM^ plus 3 % sucrose, and kept in dark at 23 ± 2 °C.

### Callus induction

Callus was obtained from *in vitro* germinated *C. asiatica* seedling using the protocol reported by Loc and An with slight modifications^15^. Briefly, about 1 cm long stolon explants of 15 days old seedling of *C. asiatica* were cut and inoculated on MS medium (pH 5.7) supplemented with 1 mg/L of 1-naphthaleneacetic acid, 1 mg/L of 6-benzylaminopurine, 0.5% Agargellan^TM^ and 3% sucrose. The cultures were incubated at 23 ± 2 °C under 16 h photoperiod and 30 µE m^−2^ s^−1^ irradiance provided by cool white fluorescent tubes. Callus were routinely sub-cultured onto the same medium every 30 days.

### Preparation of extracts and standards

About 100 mg of ground callus and authentic plant samples were individually mixed with 1 mL of methanol, 95% ethanol, 75% ethanol or 50% ethanol (v/v); extracted for 30 min by using sonication at 25 °C; and then filtered through Whatman Grade 42 paper (Whatman plc; Maidstone, UK). The filtrate was then used for HPTLC and HPLC analysis.

For standards (asiaticoside, asiatic acid, madecassoside, madecassic acid, kaempferol, quercetin and rutin), the working solutions were prepared in methanol at final concentration of 1 mg/mL and filtered for further use.

### High-performance thin-layer chromatography (HPTLC)

The compounds in extracts were separated by using HPTLC technique. Briefly, 5 µL/band of the Centella extracts and 4 µL/band of the standards were applied on the Merck silica gel 60 F_254_ glass HPTLC plates (20 × 10 cm) by a semi-automatic sampler Linomat 5 (Camag; Muttenz, Switzerland) using 8 mm bands, 11.4 mm apart, 8 mm from the lower edge, and 20 mm from the left plate edge. The plates were developed in the chamber saturation containing mobile phases. For separation of triterpenoids, the mobile phase was dichloromethane: methanol: water (14:6:1, V/V/V)^16^. For separation of flavonoids, ethyl acetate: methanol: water (100:25:10, V/V/V) was used as mobile phase. The development was done with a migration distance of 70 mm using an automatic development chamber ADC2 (Camag; Muttenz, Switzerland). For observation of triterpenoids, the plate was dipped with sulfuric reagent using Chromatogram Immersion Device III (Camag; Muttenz, Switzerland) and then heated at 100 °C for 3 min on the TLC Plate Heater (Camag; Muttenz,Switzerland). The derivatized plates were visualized under white light using visualizer 2 (Camag; Muttenz, Switzerland). For detection of flavonoids, the chromatogram was heated at 110 °C for 3 min and then was dipped in NP/PEG reagent. The derivatized plates were visualized under UV light at 366 nm using visualizer 2 (Camag; Muttenz, Switzerland).

### HPTLC-DPPH bioautographic assay

In order to estimate the antioxidant activity of tested samples, HPTLC-DPPH was performed as a screening assay. After development, the HPTLC plates were immersed with 0.02% DPPH-methanol solution and incubated in dark for 2 h^17^. The chromatogram was then examined under white light. Generated bands with yellowish color on the purple backgrounds were considered as compounds with antioxidant activities.

### DPPH assay

The radical scavenging activities of tested extracts were evaluated by traditional DPPH assay^18^. Briefly, 100 µL of DPPH solution (0.008% w/v DPPH in methanol) was mixed with 100 µL of varying concentrations of callus extract in a 96-well microplate and incubated at 25 °C for 30 min. After incubation, the absorbance was recorded at 517 nm using CLARIOStar microplate reader (BMG Labtech; Ortenberg, Germany). 100% methanol was used as a control.

The percentage of radical scavenging activity (%RSA) of sample was calculated using the following formula:

$$\% ~RSA = ~\frac{{\left( {~OD517_{{Control}} - ~OD517_{{sample}} } \right) \times ~100}}{{OD517_{{Control}} }}$$

OD517_Control_ = Optical density at 517 nm of 100% methanol; OD517_sample_ = Optical density at 517 nm of samples.

The concentration of extracts that resulted in 50% RSA was estimated from dose-response curve using GraphPad Prism version 9.0 (GraphPad Software).

### HPLC analysis

Seven standards, namely, asiaticoside, asiatic acid, madecassoside, madecassic acid, kaempferol, quercetin and rutin, along with the callus extract were used for the analysis. The analysis was performed using a Shimadzu HPLC (LC-20A) connected with a PDA detector (Shimadzu; Kyoto, Japan). A C-18 column (100 mm × 4.6 mm, 5 µm particle size, Phenomenex, USA) was used. The HPLC analysis was conducted according to the method of Buraphaka and Putalun^19^. The mobile phase used was a gradient of 1% acetic acid (A) and acetonitrile (B) at a flow rate of 1 mL/min with the following linear gradient HPLC solvent program: solvent B was increased from 0 to 25% over 20 min and further increased to 40% over 35 min, followed by further increase to 65% till 40 min and then held at 100% A for 5 min before returning to the initial state. The column temperature was controlled at 30 °C, and chromatograms were recorded at 210 and 320 nm.

### Cell culture

Human foreskin fibroblasts, BJ (ATCC^®^ CRL-2522), were obtained from American Type Culture Collection (ATCC; Manassas, VA, USA). The condition for cell culture was followed the standard protocol as described previously^20^. BJ cells were maintained in MEM supplemented with 1 mM pyruvate, 10% FBS, and 100 U/mL penicillin and 100 µg/mL streptomycin. The cells were cultured at 37 °C in a humidified atmosphere with 5% CO_2_.

### Exposure of H_2_O_2_

H_2_O_2_ was used as an inducing agent for oxidative stress^18^. A solution of H_2_O_2_ was freshly prepared in serum-free MEM prior each experiment. To examine the cytotoxicity of H_2_O_2_, the BJ cells were exposed to H_2_O_2_ at 200 µM for 1 h. For a control group, the medium was replaced with a fresh serum-free MEM similar to the H_2_O_2_-treated cells.

### Centella extract treatments

The 50% ethanolic extracts of *Centella* were dissolved with DMSO to produce a 1 mg/mL stock solutions. The treatment protocol was slightly modified from our previous *in vitro* experiments on skin cells^21,22^. To observe the preventive activities of *Centella* extracts against H_2_O_2_-induced cytotoxicity, BJ cells were pre-treated with the *Centella* extracts (final concentration, 15–60 µg/mL; 0.5% DMSO) for 24 h prior to H_2_O_2_ treatment. Control groups were incubated with an equivalent amount of DMSO (0.5% DMSO). DMSO at 0.5% is considered as the non-toxic concentration of DMSO for the BJ cells.

### Cell viability assay

The cell viability was determined by using MTT assay^23^. Briefly, BJ cells were seeded into 96-well plates at density of 20,000 cells/well. The cells were cultured for 24 h at 37 °C, 5% CO_2_ before exposure to the experimental condition. After indicated treatments, exposure media were aspirated, and the cells were washed with phosphate buffer solution (PBS; pH 7.4). The washed cells were incubated in dark with MTT solution (200 µL; 1 mg/mL in serum-free MEM). After 3-h incubation, the MTT solution was removed, and the formazan crystals were dissolved with DMSO (200 µL/well). The absorbance of the formazan solution was then measured at 570 nm using the CLARIOStar microplate reader.

### Measurement of an intracellular ATP

The BJ cells were seeded into multiple culture dishes (60 mm) at a density of 250,000 cells per dish in 4 mL of the MEM. Cells were cultured for 24 h at 37 ºC, 5% CO_2_ before exposure to experimental conditions. Following the indicated treatments, treated cells were trypsinized and resuspended with PBS. Levels of intracellular ATP were immediately measured with CellTiter-Glo Luminescent Cell Viability Assay (Promega, Madison, WI, USA; Cat no. G7570) as previously described^24^. Briefly, a cell suspension (50,000 cells in 100 µL of PBS) was added into each well of opaque walled, 96-well white microplate. Subsequently, 100 µL of the CellTiter-Glo reagent was homogenously mixed with the cell suspension at room temperature to induce cell lysis and start the luminescent reaction. After a 10-min incubation, the luminescent signal was measured using the CLARIOStar microplate reader. The generated luminescent signal is directly proportional to the amount of ATP in the sample. To estimate the intracellular level of ATP, standard curves for each experiment were produced with serial dilutions of ATP solutions (0–1000 μM). The amount of ATP in each sample was calculated from the corresponding standard curve and then further transformed to an intracellular concentration using the cell number.

### Real time-quantitative polymerase chain reaction (RT-qPCR)

After treatments, total RNA was isolated from BJ cells by using GENEzol reagent (Geneaid Biotech Ltd., New Taipei City, Taiwan; Cat no. GZR100) according to manufacturer’s protocol. One microgram of extracted RNA was then reverse transcribed into cDNA by using RevertAid First Strand cDNA Synthesis Kit (Thermo Fisher Scientific, Waltham, MA, USA; Cat no. K1621). The RT-qPCR was carried out in CFX96 Touch Real-Time PCR Detection System (Bio-Rad Laboratories, Hercules, CA, USA) with Luna Universal qPCR Master Mix (New England Biolabs, Ipswich, MA, USA; Cat No: M3003L) following the manufacturer’s instruction. The expression of interested genes were quantified by using ΔΔCq approach with normalization to house-keeping *β-Actin*. The conditions for RT-qPCR were modified from previous study by Biagini and colleagues^25^. The PCR conditions, sequences of primer nucleotide (Macrogen, Seoul, South Korea) and analysis of RNA extraction are listed in Supplementary Table S1, S2, and S3 respectively.

### Statistical analysis

The unpaired *t* test and one-way analysis of variance (ANOVA) with Tukey’s *post hoc* were conducted to evaluate statistical differences between means. All means and standard errors of the mean (SEMs) were calculated from three independent experiments. Data were expressed as the mean ± SEM. Statistical analysis was performed using GraphPad Prism version 9.0.

## Results

### Antioxidant activities of Centella extracts

**Figure 1:**
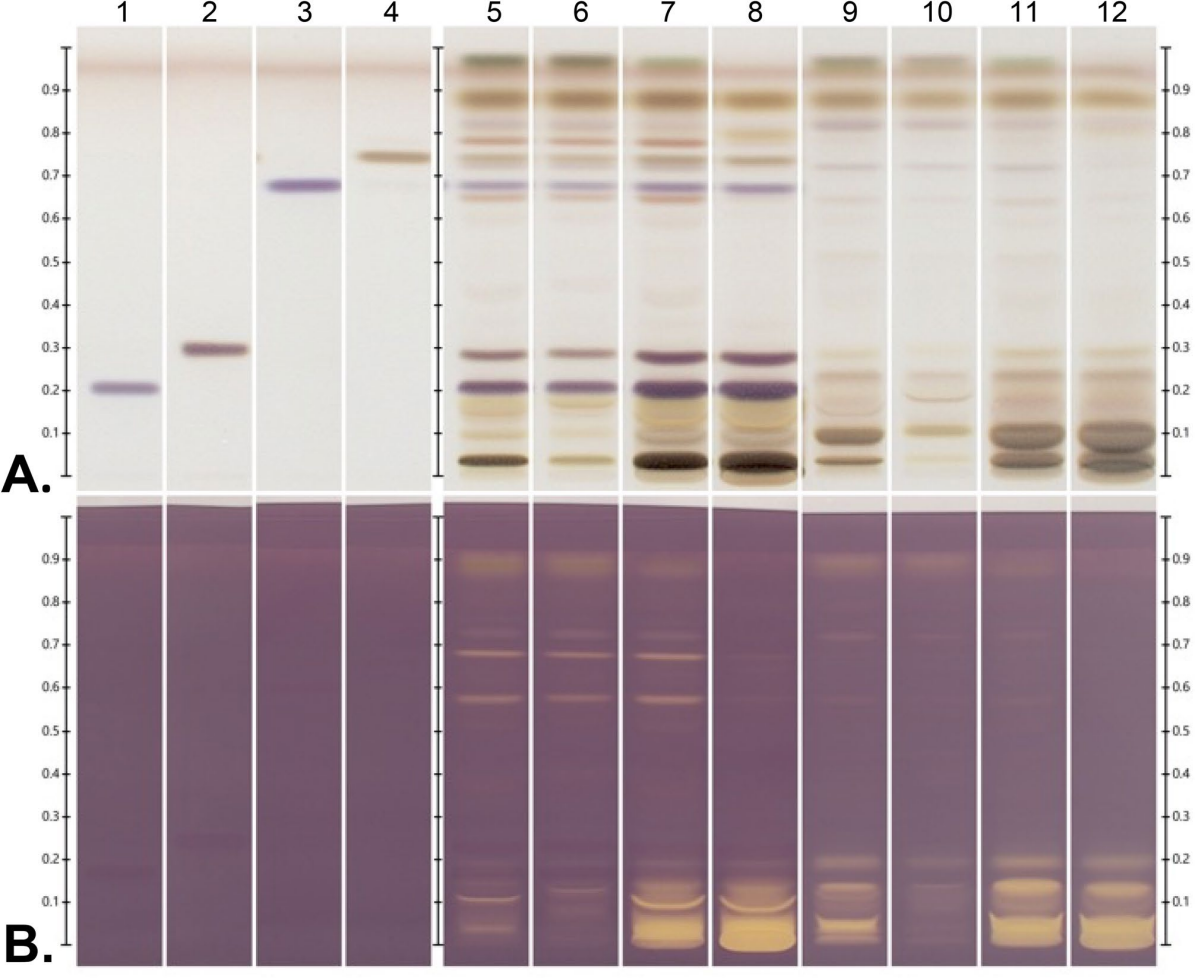
The antioxidant activities of *Centella* extracts are not due to asiaticoside, asiatic acid, madecassoside and madecassic acid. HPTLC fingerprints of triterpene standards (**1**, madecassoside; **2**, asiaticoside; **3** madecassic acid; **4**, asiatic acid), APE (**5**, methanol; **6**, 95 % ethanol; **7**, 70 % ethanol; **8**, 50 % ethanol), and CE (**9**, methanol; **10**, 95 % ethanol; **11**, 70 % ethanol; **12**, 50 % ethanol). (**A**) The HPTLC plate was derivatized with anisaldehyde sulphuric acid reagent and visualized with white light. (**B**) The HPTLC plate was dipped with DPPH reagent and visualized with white light.

To determine a suitable solvent for extraction process, callus culture and authentic plant of *C. asiatica* were extracted with series of solvents, including methanol, 95% ethanol, 70% ethanol, and 50% ethanol. Due to their abundance and pharmacological activities, asiaticoside, asiatic acid, madecassoside, and madecassic acid are considered to be major active components of *C. asiatica*^7,26^. To examine whether callus extract (CE) and authentic plant extract (APE) contains these triterpenoids, active compounds in extracts were separated with HPTLC approach. All of four centelloids were observed in APE (Lanes 5–8; Fig. 1A); however, none of them were detected in CE (Lanes 9–12; Fig. 1A). To screen active antioxidants in the extracts, HPTLC plates were further derivatized with DPPH reagent. A stable free radical DPPH is a widely used substrate to evaluate free radical scavenging activities of tested compounds. On HPTLC plates, the compounds generating yellow zones against the purple background were considered as antioxidants. The CE and APE exhibited strong free radical scavenging activities. Interestingly, all of tested triterpenoids did not show antioxidant activities (Fig. 1B; Lanes 1–4). The CE showed significant antioxidant activity in the absence of these standards. These HPTLC-DPPH data suggested that asiaticoside, asiaticoside, asiatic acid, madecassoside, and madecassic acid do not contribute to antioxidant properties of the extracts.

**Figure 2:**
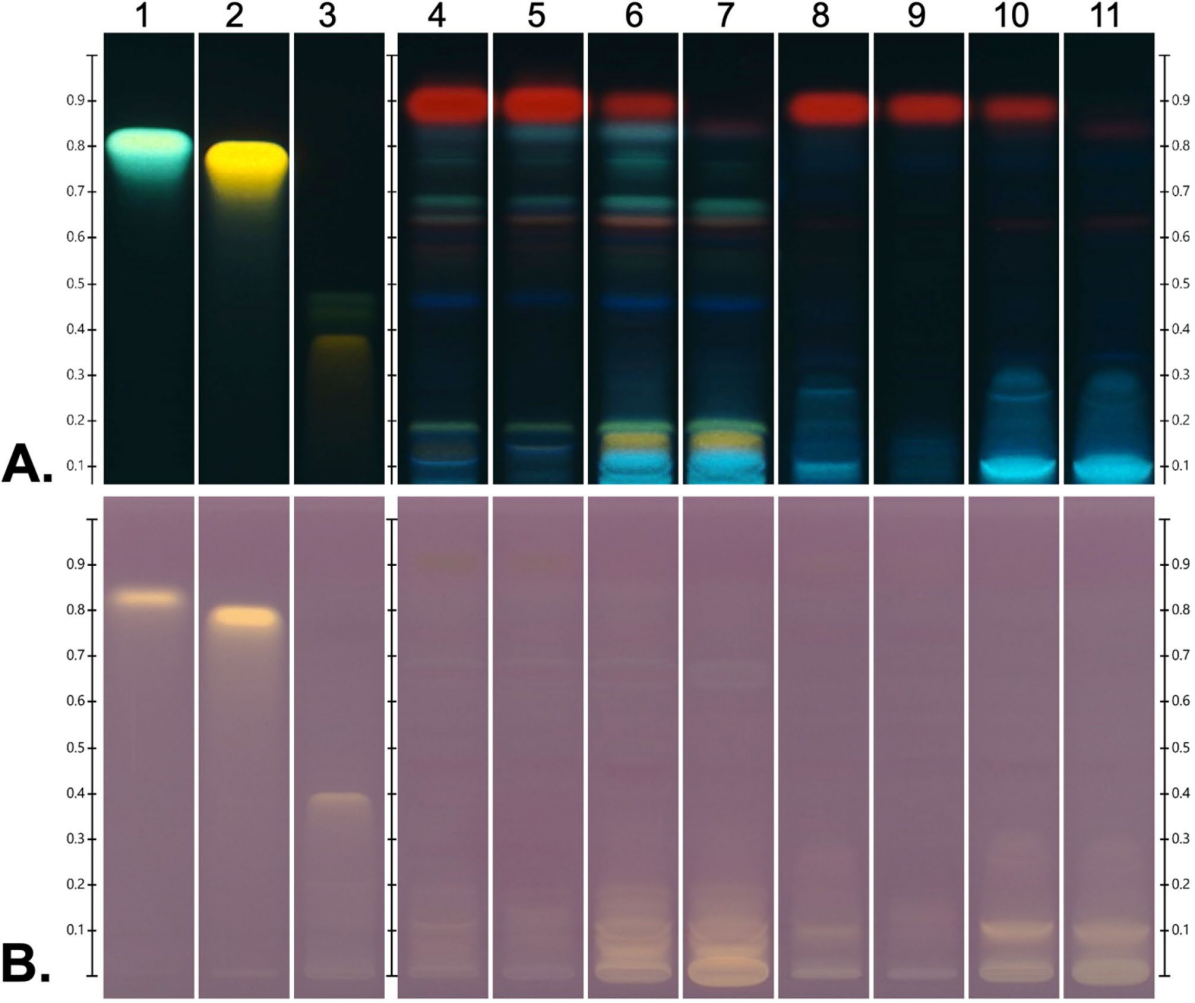
The antioxidant activities of *Centella* extracts are not due to kaempferol, quercetin and rutin. HPTLC fingerprints of flavonoid standards (**1**, kaempferol; **2**, quercetin; **3**, rutin), APE (**4**, methanol; **5**, 95 % ethanol; **6**, 70 % ethanol; **7**, 50 % ethanol), and CE (**8**, methanol; **9**, 95 % ethanol; **10**, 70 % ethanol; **11**, 50 % ethanol). (**A**) The HPTLC plate derivatized with NP/PEG solution and visualized under UV light at 366 nm. (**B**) The HPTLC plate was immersed with DPPH reagent and visualized with white light.

Kaempferol, quercetin, and rutin reported in *C. asiatica*^27,28^ are flavonoid compounds with antioxidant activities. It was suspected that these compounds may contribute to the antioxidant activity of the extracts. However, the chromatogram on the HPTLC plate demonstrated that these three flavonoids were not presented in any extracts (Fig. 2A). The data from HPTLC-DPPH showed that CE and APE exhibited significant antioxidant activities, as evidenced by a great intensity of yellow spots (Fig. 2B). Moreover, *Centella* extracts exhibited potent radical scavenging properties without the presence of kaempferol, quercetin and rutin (Fig. 2B). Taken together, these results strongly suggested that antioxidant activities of extracts are not due to these three flavonoids but possibly from other flavonoids.

As demonstrated in Figs. 1B and 2B, it was observed that 70% and 50% ethanolic extracts of both authentic plant (Fig. 1B, lanes 7 and 8; Fig. 2B, lanes 6 and 7) and callus (Fig. 1B, lanes 11 and 12; Fig. 2B lanes 10 and 11) have the strongest intensity of bands in HPTLC-DPPH analysis. Hence, 50% ethanol would be the suitable solvent to be used in extraction of *C. asiatica* because (1) 50% ethanolic extracts of callus and authentic plant exhibit high antioxidant activity; (2) the amount of organic solvent is less than 70% ethanolic extracts; and (3) ethanol is safer than methanol, then it is practical to develop product for cosemeceutical purposes. Hence, 50% ethanolic extracts of callus and authentic plant were chosen for further experiments of this study.

As shown in Figs. 1 and 2, the antioxidant properties of CE were comparable to APE. We then further evaluate total antioxidant activity of these two 50%-ethanolic extracts by determination of IC50 of %RSA with DPPH assay. The IC50 of %RSA represents the concentration of extracts that is required for 50% free radical scavenging activity. We exhibited that CE had approximately 2.5 folds greater ability to neutralize free radicals than APE (Supplementary Fig. 1; CE, 98.6 ± 1.6 µg/mL vs APE, 243.3 ± 14.6 µg/mL). These results suggest that extract from callus culture possesses a strong free radical scavenging ability, which is even greater than from native plant.

### HPLC analysis of Centella extracts

**Figure 3:**
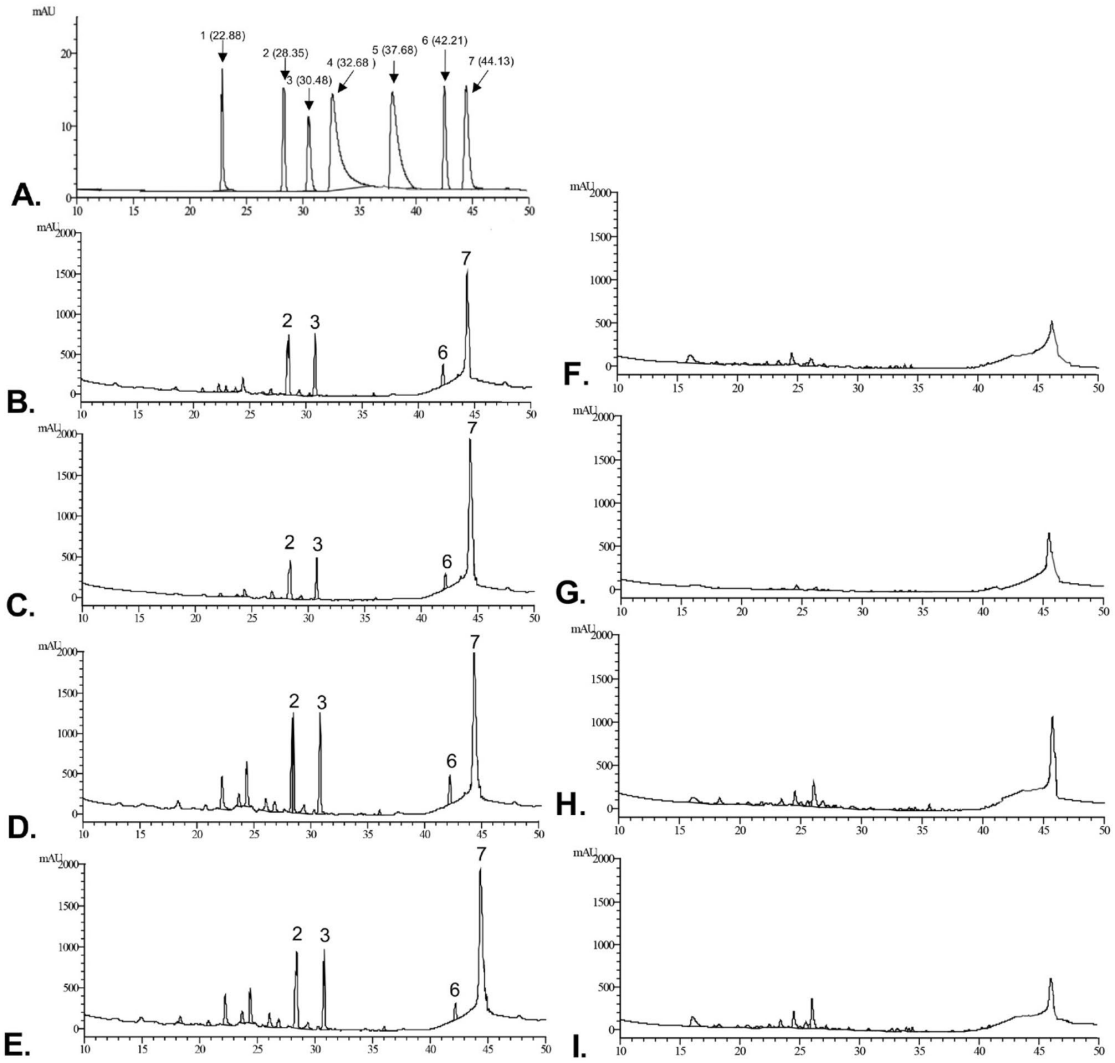
*Centella* extract from callus culture has unique chemical profiles. HPLC chromatogram of (**A**) standard solutions of triterpenes and flavonoids (**1**, rutin; **2**, madecassoside; **3**, asiaticoside; **4**, quercetin; **5**, kaempferol; **6**, madecassic acid; **7**, asiatic acid); (**B–E**) Authentic plant extract (**B,** methanol; **C,** 95% ethanol; **D,** 70% ethanol, **E**, 50% ethanol); and (**F–I**) Callus extract (**F,** methanol; **G,** 95% ethanol; **H,** 70% ethanol, **I**, 50% ethanol). The retention time (RT) was shown in parenthesis.

HPLC was conducted to analyze and compare chemical compositions in *Centella* extracts (Fig. 3) and validate the result of HPTLC technique. On comparison with retention time (RT) between standards and extracts, none of CE contained asiaticoside (RT = 30.48 min), asiatic acid (RT = 44.13 min), madecassoside (RT = 28.35 min), madecassic acid (RT = 42.21 min), kaempferol (RT = 37.68 min), quercetin (RT = 32.68 min), and rutin (RT = 22.88 min). All the standards were detected in the APE, irrespective of the solvent used for extraction. These HPLC data are consistent with HPTLC results demonstrated in Figs. 1 and 2. Moreover, CE demonstrated different chemical profiles when compared to APE. Hence, the difference in antioxidant capacity between CE vs APE could possibly be due to variation in active components.

### Protective effects of Centella extracts against oxidative damage on human fibroblasts

**Figure 4:**
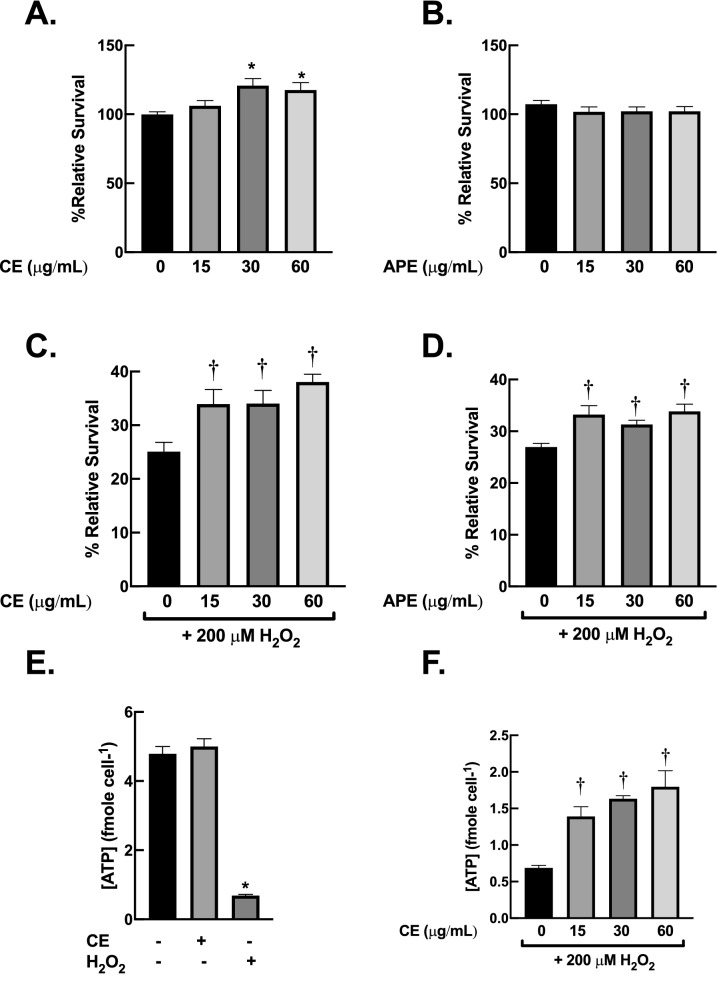
*Centella* extracts prevent H_2_O_2_-induced cytotoxicity in dermal fibroblasts. (**A**) and (**B**) CE and APE at 15–60 µg/mL did not cause toxicity in dermal fibroblasts. BJ cells were incubated with CE or APE (15–60 µg/mL) for 24 h. (**C**) and (**D**) CE and APE inhibit oxidative damage from H_2_O_2_ exposure. BJ cells were pre-incubated with CE or APE (15–60 µg/mL) for 24 h then exposed to H_2_O_2_ (200 µM) for 1 h. The cell viability was evaluated with MTT assay immediately after the treatments. (**E**) and (**F**) CE inhibit depletion of intracellular ATP following H_2_O_2_ exposure. The protocol for treatment was as (**A**) and (**C**). The amounts of ATP were measured immediately after treatments (*n* = 3; mean ± SEM; *P < 0.05 vs untreated control; ^†^*P* < 0.05 vs H_2_O_2_-treated cells).

The compounds with antioxidant capacity have been proposed as promising candidate for anti-skin-aging agents^29^. According to cell-free based antioxidant assay, CE and APE were selected to further examine their beneficial activities on human dermal fibroblast BJ cells. Firstly, we evaluated potential toxicity of CE and APE on dermal fibroblasts. The data from MTT assay demonstrated that 24-h exposure of CE or APE, up to 60 µg/mL, did not cause toxicity to BJ cells (Fig. 4A, B). Hence, concentrations of CE and APE at 15–60 µg/mL were chosen for subsequent experiments.

To investigate the preventive effects of CE and APE against oxidative insults, BJ cells were pre-treated with CE or APE at non-toxic concentration for 24 h prior to H_2_O_2_ treatment. Exposure to H_2_O_2_ (200 µM; 1 h) reduced cell viability of BJ cells to approximately 25% (Fig. 4C). Pre-treatment with CE significantly inhibited H_2_O_2_-induced toxicity (Fig. 4C). Interestingly, the protective effects of CE were comparable to APE pre-treatments (Fig. 4D). These data suggested that CE and APE could exert their beneficial effects against H_2_O_2_ on dermal fibroblasts *via* their antioxidant activities.

The imbalance of cellular bioenergetics due to oxidative stress is closely linked to aging processes of several tissues, including skin^30^. Exposure to H_2_O_2_ caused an immediate depletion of steady-state levels of ATP in dermal fibroblasts (Fig. 4E). In contrast, treatment with non-toxic concentration of CE (60 µg/mL; 24 h) did not alter intracellular levels of ATP, supporting the safety of CE on the selected concentration on BJ cells (Fig. 4E). Interestingly, pre-treatment with CE significantly inhibited reduction of intracellular ATP pool following H_2_O_2_ treatment in dose-dependent fashion (Fig. 4F). The results from these bioenergetics studies were consistent with the protective effects of CE against oxidative damage from MTT assay, highlighting the contribution of antioxidant activity of CE on its protective effect against oxidative insults.

### *Effects of Centella extracts on* expression of antioxidant enzymes

**Figure 5:**
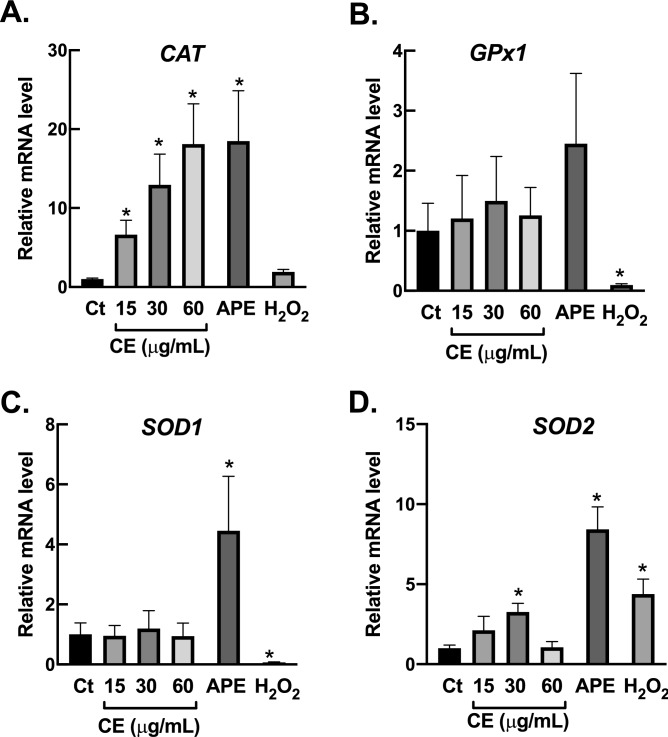
*Centella* extracts elevate transcription of antioxidant enzymes. (**A**–**D**) CE promoted *CAT* expression, while APE enhanced *SOD1* and *SOD2* expression. BJ cells were incubated with CE (15–60 µg/mL; 24 h) or APE (60 µg/mL; 24 h) or H_2_O_2_ (200 µM; 1 h). The expressions of antioxidant machineries, including *CAT*, *GPx1*, *SOD1*, and *SOD2*, were determined with RT-PCR following treatments (*n* = 3; mean ± SEM; *P < 0.05 vs untreated control).

The beneficial effects of CE and APE against oxidative damage as showed in Fig. 4 are possibly due to an induction of capacity of fibroblasts to detoxify reactive oxygen species (ROS). To examine this hypothesis, the mRNA expression of key antioxidant enzymes, including catalase (CAT), glutathione peroxidase 1 (GPx1), superoxide dismutase 1 (SOD1), and superoxide dismutase 2 (SOD2), were measured following 24-h treatments. Our RT-qPCR results demonstrated that CE and APE have different profiles on the induction of antioxidant machinery of fibroblasts; CE induced expression of *CAT* in dose-dependent fashion (Fig. 5A), while APE enhanced expression of both *SOD1* (Fig. 5C) and *SOD2* (Fig. 5D). The expression of *GPx1* did not alter with either CE or APE treatment (Fig. 5B). These RT-qPCR data suggested that an upregulation of cellular antioxidant enzymes is the principal factor for protective effects of CE and APE.

**Figure 6:**
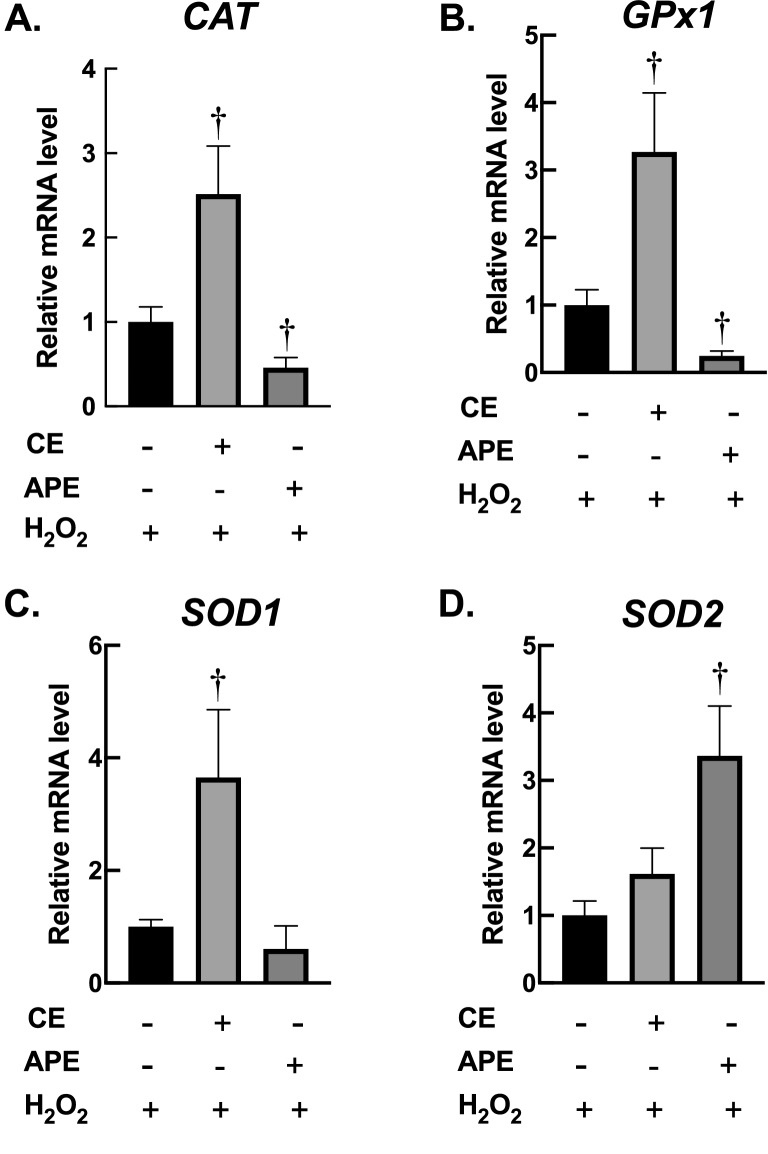
*Centella* extracts promote expression of antioxidant enzymes following H_2_O_2_ treatment. (**A**–**D**) In response to H_2_O_2_ treatment, *CAT*, *GPX1* and *SOD1* expressions were increased in CE-treated fibroblasts, while *SOD2* transcription were elevated in APE-treated fibroblasts. BJ cells were incubated with CE (60 µg/mL; 24 h) or APE (60 µg/mL; 24 h), then exposed to H_2_O_2_ (200 µM; 1 h). The transcriptional levels of *CAT*, *GPx1*, *SOD1*, and *SOD2*, were evaluated with RT-PCR following treatments (*n* = 3; mean ± SEM; ^†^*P* < 0.05 vs H_2_O_2_-treated cells).

We also observed the effects of *Centella* extracts on expression of antioxidant enzymes after H_2_O_2_ treatment. Exposure to H_2_O_2_ leaded to dramatic decrease in *GPx1* (Fig. 5B) and *SOD1* (Fig. 5C); and significant increase in *SOD2* (Fig. 5D). The increase of *SOD2* expression is possibly due to adaptive mechanism of fibroblasts following H_2_O_2_ treatment. This phenomenon has been reported in several models of aged fibroblasts^31–33^. Again, CE and APE demonstrated distinctive effects on induction of antioxidant enzymes following H_2_O_2_ exposure. Pre-treatment with CE induced *CAT* (Fig. 6A), *GPx1* (Fig. 6B) and *SOD1* expression (Fig. 6C), whereas APE upregulated *SOD2* expression following 1-h exposure to H_2_O_2_ (Fig. 6D). Altogether, these results clearly exhibited that *Centella* extracts could prevent H_2_O_2_ cytotoxicity by an enhanced capacity of fibroblasts to remove deleterious ROS.

### *Effects of Centella extracts on *expression of matrix metalloprotease 9 (*MMP-9*)

**Figure 7:**
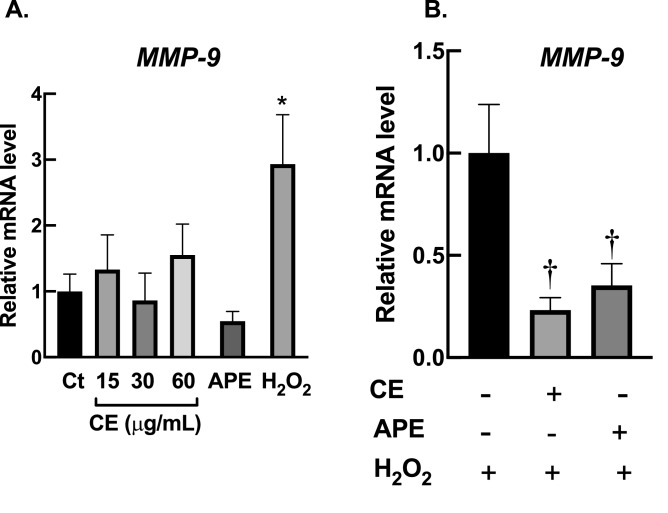
*Centella* extracts inhibit expression of *MMP9* following H_2_O_2_ treatment. (**A**) Exposure to H_2_O_2_ resulted in elevation of *MMP9* expression, while CE and APE did not affect the transcription of *MMP9*. BJ cells were treated CE (15–60 µg/mL; 24 h) or APE (60 µg/mL; 24 h) or H_2_O_2_ (200 µM; 1 h). (**B**) CE and APE suppressed H_2_O_2_-induced *MMP9* expression. BJ cells were pre-incubated with CE or APE (60 µg/mL) for 24 h then exposed to H_2_O_2_ (200 µM) for 1 h. The transcriptional levels of *MMP9* were evaluated with RT-PCR following treatments (*n* = 3; mean ± SEM; *P < 0.05 vs untreated control; ^†^*P* < 0.05 vs H_2_O_2_-treated cells).

Matrix metalloprotease-9 (MMP-9) is the gelatinase enzyme that is responsible for regulation of homeostasis of collagen. Formation of oxidative stress can lead to upregulation of MMP-9 in fibroblasts, which subsequently results in degradation of collagen. The increased breakdown of collagen by MMP-9 appears to be the major factor for aging processes of skin tissue^34,35^. Here, we demonstrated that exposure to H_2_O_2_ markedly upregulated the expression level of *MMP-9* of dermal fibroblasts (Fig. 7A). Elevation of *MMP-9* expression was significantly suppressed by CE and APE pre-treatment (Fig. 7B). These data suggested that CE and APE may possess anti-aging activities *via* an inhibition of *MMP-9* transcription.

## Discussions

*C. asiatica* is a medicinal plant with broad range of pharmacological effects e.g. antioxidant^8–11^, anti-inflammation^36,37^, wound healing^38–40^, neuroprotective^8,9^ and memory improvement^41,42^. Due to its high therapeutic potential, the demand for this plant in cosmeceutical and pharmaceutical industries has exceeded the supply from conventional cultivation. Callus culture offers a manufacturing system which ensures the continuous supply of compounds with uniform quality and high yields^13^. Here, we demonstrated that 50% ethanol is the suitable solvent to be used in extraction process for *C. asiatica* due to (i.) antioxidant activity of extracts; and (ii.) safety from reduced solvent residue. The 50% ethanolic extracts from callus culture has unique chemical profiles and biological activities. Compared to APE, the data from HPTLC (Fig. 1,2) and HPLC (Fig. 3) suggested that (1) CE comprises of unique antioxidant compounds which should be further identified; (2) the types of secondary metabolites in CE are different from APE. Interestingly, results from DPPH assay showed that the total antioxidant activity of CE is higher than APE (Supplementary Fig. 1). The greater antioxidant activity of CE is possibly due to the differences in active components in the extracts. We demonstrated that the major triterpenoids of *C. asiatica* (asiaticoside, asiaticoside, asiatic acid, madecassoside and madecassic acid) are not responsible for antioxidant activities of CE (Fig. 1). These findings are parallel to previous observation demonstrating that triterpene-free-extract of *C. asiatica* possesses greater *in vitro* radical scavenging properties as well as *in vivo* antioxidant activities than triterpene-enriched-extract. Triterpene-free-fraction of *C. asiatica* has significantly stronger in free-radical scavenging activities than triterpene-enriched-extract as determined by several *in vitro* anti-radical assays, including DPPH, ABTS, NORAC, ORAC and NO scavenging assays. Moreover, pre-treatment with triterpene-free-extract of *C. asiatica* reduced levels of malonaldehyde, an oxidative stress marker, in brain tissues of scopolamine-treated-rat, while triterpene-enriched-fraction did not have these protective effects^43^. These preclinical findings support our results that the centelloids are not principal contributors for antioxidant activity of *C. asiatica*.

Kaempferol, quercetin, and rutin have been reported to be major flavonoids with antioxidant properties of *C. asiatica*^27,28^. Our HPTLC-DPPH and HPLC results demonstrated that these flavonoids did not present in 50% ethanolic extracts from both callus culture and authentic plant; then these series of flavonoids did not contribute to antioxidant activities of these extracts (Fig. 2). Hence, future studies are required to identify and characterize novel antioxidants presented in these extracts.

The process of skin aging is closely associated with an induction of oxidative stress in dermal fibroblasts^1^. Hence, compound that can prevent oxidative damage in dermal fibroblasts could be a potential candidate to be used as anti-skin-aging in cosmeceutical product. We then further investigated biological activities of *Centella* extracts against oxidative stress on dermal fibroblasts. Our data from MTT assay as well as bioenergetic study showed that CE significantly inhibited the cytotoxicity of H_2_O_2_ on dermal fibroblasts (Fig. 4). The preventive effects of CE were comparable to APE. Moreover, we found that the protective activities of these extracts could be due to an increase in capacity of fibroblasts to eliminate ROS. We used RT-qPCR approach to investigate the alterations of mRNA expression of key antioxidant enzymes in response to treatments. Catalase is the major enzyme involved in the elimination of high fluxes of H_2_O_2_, while GPx1 is responsible for detoxification of low fluxes of H_2_O_2_^44^. SOD1 is a cytosolic Cu/Zn-superoxide dismutase, whereas SOD2 is a mitochondrial Mn-superoxide dismutase. Both SOD1 and SOD2 are crucial for removal of superoxide free radical (O_2_^·−^) during oxidative stress condition. The dismutation of O_2_^·−^ by SOD leads to generation of H_2_O_2_, which in turn eliminated by catalase and other peroxidases^45^. RT-qPCR data revealed that CE and APE have different downstream targets on cellular antioxidant machineries. Treatment with CE promoted mRNA expression of H_2_O_2_-detoxifying enzyme, *CAT*. Supplementation with APE enhanced mRNA expression of O_2_^·−^-removal enzymes, *SOD1* and *SOD2* (Fig. 5). In response to H_2_O_2_ treatment, pre-exposure to CE induced *CAT*, *GPx1* and *SOD1* expression, whereas pre-treatment with APE upregulated *SOD2* expression (Fig. 6). Upregulation of these antioxidant machineries due to *Centella* extracts could promote capacity of fibroblasts to eliminate harmful ROS, resulting in suppression of oxidative damage and prevention of cell death. Further studies on protein levels and enzymatic activities of antioxidant machineries following treatments of CE and APE are necessary to better understand the protective mechanisms of these extracts on fibroblasts.

Consistent with our findings, many preclinical studies also demonstrated that the beneficial activity of *Centella* extracts against oxidative-stress-related-disorders could be due to an augmentation in expression and/or activity of cellular antioxidant enzymes. In rat model of hepatic injury, administration of *Centella* extract prevented hepatotoxicity through an increased level of catalase, SOD and GPx in liver tissues^46^. In hamster model of hyperlipidemia, supplementation with ethanolic extract of *C. asiatica* promote hepatic function by an enhanced expression of SOD and GPx in hepatic tissues^47^. In diabetic rat, aqueous extracts from *C. asiatica* ameliorate hippocampal dysfunction by an induction of catalase, SOD and GPx expression in hippocampus^48^. In stroke model, supplementation with ethanolic extract of *C. asiatica* prevented brain injury through a restoration of glutathione level and augmentation of catalase, SOD and GPx activities in ischemic rat^49^. Taken together, these previous observations strongly support our RT-QPCR results which demonstrating that 50% ethanolic extracts of *Centella* inhibited oxidative damage on human dermal fibroblasts via an upregulation of antioxidant enzymes.

The nuclear factor erythroid 2-related factor (Nrf2)/ antioxidant response element (ARE) pathway is an essential signaling network that maintains redox homeostasis for human cells^50^. Nrf2 functions as a redox sensor for oxidative stress. In the presence of oxidative insults, Nrf2 translocates into nucleus and binds to promoter regions of ARE, leading to transcriptional activation of a battery of cytoprotective and detoxification genes, including *CAT*, *GPx* and *SOD*^51–54^. Activation of Nrf2/ARE pathway by *Centella* extracts has been observed in preclinical models of oxidative-stress-related disease. A water extract of *Centella asiatica* can attenuate oxidative stress and activate Nrf2/ARE network in isolated primary neurons and rodent models of pathological cognitive impairment. The activation of this pathway by *Centella* extract is strongly accompanied by an improvement in neuronal health and cognitive function^8,55–58^. Recently, Park and colleagues demonstrated that pre-treatment with *Centella* extract can prevent the progression of age-related macular degeneration *in vitro* and *in vivo*. They clearly showed that the cytoprotective activities against oxidative damage of *Centella* extract in cell culture and animal experiment is mainly due to the regulation of Nrf2 pathway^59^. Hence, we postulated that Nrf2/ARE pathway could be a potential mediator for the beneficial effects of Centella extracts in our model. Further pharmacological study is required to validate this hypothesis.

**Figure 8:**
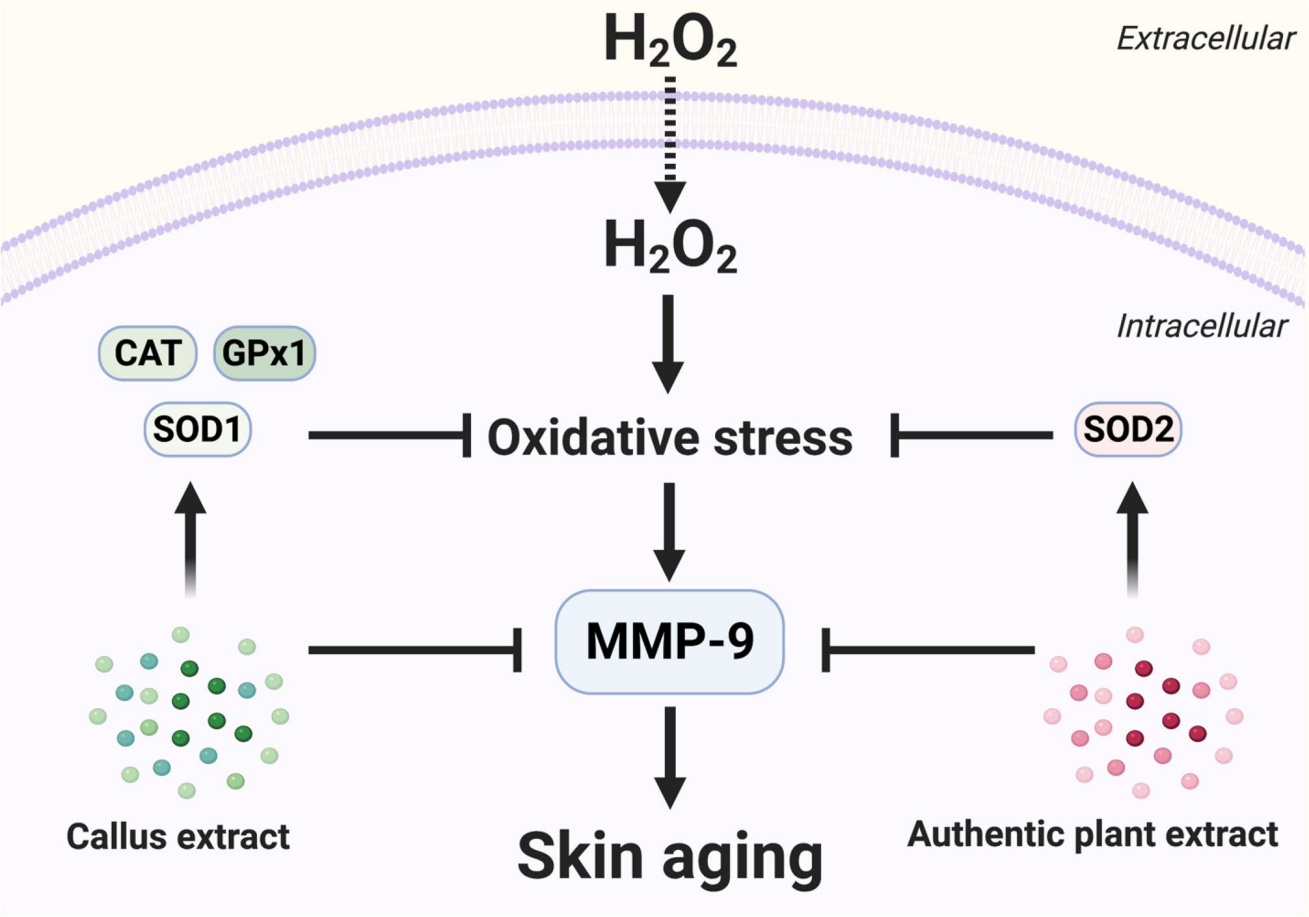
Proposed mechanisms for antioxidant and anti-skin aging activities of *Centella* extract. Pre-treatment with *Centella* extracts prevent H_2_O_2_-induced cytotoxicity on dermal fibroblasts by upregulation of cellular antioxidant machineries. Supplementation with CE upregulated *CAT*, *GPx1* and *SOD1* expression, whereas pre-treatment with APE induced *SOD2* expression following H_2_O_2_ treatment. Moreover, pre-treatment with CE or APE suppressed H_2_O_2_-mediated-upregulation of *MMP-9*. This figure was created with BioRender.com.

In addition to effects on cellular antioxidant enzymes, we also observed activities of *Centella* extracts on expression of *MMP-9*. MMP-9 is zinc-containing-gelatinase which plays an important role in degradation of dermal extracellular matrix, especially collagen type IV. Environmental insults, e.g. UV irradiation, tobacco smoke, and pollutants, have been reported to induce expression of *MMP-9* of skin cells through formation of oxidative stress^35^. Upregulation of *MMP-9* leads to fragmentation of dermal collagen; thereby diminish skin elasticity and integrity; and eventually promote wrinkle and sagging formation of skin^34,60^. Hence, agents with MMP-9 inhibitory activities would be an attractive candidate to combat skin aging^61^. Our RT-qPCR data demonstrated that CE and APE significantly inhibited upregulation of *MMP9* following H_2_O_2_ exposure (Fig. 7), suggesting anti-skin-aging activities of these extracts. Future observations on detailed mechanisms of CE/APE-mediated-*MMP9*-expression are required to better understand their anti-skin-aging effects. The proposed mechanisms for antioxidant and anti-skin aging activities of CE and APE are summarized in Fig. 8.

In conclusion, our present study provides the potential application of callus culture platform to produce biomass and biochemical from *C. asiatica*. We demonstrated that the 50% ethanolic extract from callus culture has unique chemical profiles and distinct antioxidant properties. Moreover, this study provides pharmacological evidence to support the use of *Centella* extracts as anti-skin-aging agents in cosmeceutical and pharmaceutical products.

## Supplementary information

Supplementary Information.
