# Supplementary material for: Insights into antioxidant activities and anti-skin-aging potential of callus extract from Centella asiatica (L.)
Source: Sci Rep. 2021 Jun 29;11:13459. doi: 10.1038/s41598-021-92958-7 (PMC8241881; doi:10.1038/s41598-021-92958-7)
Supplement: Supplementary file 1 — Supplementary Information. [file 41598_2021_92958_MOESM1_ESM.docx]

**Insights into Antioxidant activities and Anti-skin-aging Potential of Callus Extract from *Centella asiatica* (L.)**

Visarut Buranasudja^a^, Dolly Rani^b^, Ashwini Malla^b,c^, Khwanlada Kobtrakul^d^, Sornkanok Vimolmangkang^b,e,*^

^a^ Department of Pharmacology and Physiology, Faculty of Pharmaceutical Sciences, Chulalongkorn University, Bangkok, 10330, Thailand

^b^ Department of Pharmacognosy and Pharmaceutical Botany, Faculty of Pharmaceutical Sciences, Chulalongkorn University, Bangkok, 10330, Thailand

^c^ Research Unit for Plant-Produced Pharmaceuticals, Faculty of Pharmaceutical Sciences, Chulalongkorn University, Bangkok, 10330, Thailand

^d^Graduate Program in Pharmaceutical Science and Technology, Faculty of Pharmaceutical Sciences, Chulalongkorn University, Bangkok 10330, Thailand

^e^Research Unit for Natural Product Biotechnology, Faculty of Pharmaceutical Sciences, Chulalongkorn University, Bangkok 10330, Thailand

**Supplementary Fig. 1 *Centella* extract from callus culture has stronger radical scavenging activity than those from authentic plant.**

The %RSA of *Centella* extracts were determined using the DPPH assay. The IC_50_ of extracts required for 50% free radical scavenging activity

was calculated. The IC_50_ for the RSA of GCE was approximately 2.5-fold greater that of APE (GCE, 98.6 ± 1.6 µg/mL *v.s.* APE, 243.3 ± 14.6 µg/mL; *n* = 3; mean ± SEM, *, *p* < 0.05; RSA = radical scavenging activity)

**Supplementary Table S1** Condition of PCR amplification

| Step | Condition |
| --- | --- |
| Denaturation | 95 °C for 3 min; followed by 40 cycles of 95 °C for 5 s |
| Primer annealing | 55°C for 30 s |
| Extension | 65 °C for 5 s |

**Supplementary Table S2** The gene target and primer pairs used for RT-qPCR

| **Gene** | **Forward Primer 5’-3’** | **Reverse Primer 5’-3’** |
| --- | --- | --- |
| *β-Actin* | ACGACATGGAGAAAATCTGGCACC | ATCACGATGCCAGTGGTACGG |
| *SOD1* | AATACAGCAGGCTGTACCAGTGC | TCATGGACCACCAGTGTGCG |
| *SOD2* | ATGCAGCTGCACCACAGC | CACCACCGTTAGGGCTGAGG |
| *Catalase* | AACTGTCCCTACCGTGCTCG | ATTGGCAGTGTTGAATCTCCGC |
| *GPx1* | TCGAAGCCCTGCTGTCTCAAGG | GGTCTGGCAGAGACTGGGATCAA |
| *MMP-9* | ACTCGGGTGGCAGAGATGC | AGGTGATGTTGTGGTGGTGC |

**Supplementary Table S3** Analysis of RNA extraction

| **Samples** | **Concentration of RNA (ng/μL)** | **A260/A280** |
| --- | --- | --- |
| Untreated control | 111.8 | 2.05 |
|  | 133.2 | 1.65 |
|  | 195.8 | 1.84 |
| 15 μg/mL CE | 136.7 | 1.90 |
|  | 149.7 | 1.78 |
|  | 172.8 | 1.96 |
| 30 μg/mL CE | 167.2 | 1.86 |
|  | 123.8 | 1.81 |
|  | 143.9 | 1.89 |
| 60 μg/mL CE | 193.4 | 1.75 |
|  | 148.6 | 1.73 |
|  | 205.4 | 2.09 |
| 60 μg/mL APE | 140.2 | 1.89 |
|  | 126.1 | 1.79 |
|  | 103.6 | 1.96 |
| H_2_O_2_ | 114.9 | 1.62 |
|  | 157.9 | 2.06 |
|  | 120.5 | 1.92 |
| 60 μg/mL CE + H_2_O_2_ | 168.8 | 1.93 |
|  | 145.3 | 2.03 |
|  | 114.6 | 1.84 |
| 60 μg/mL APE + H_2_O_2_ | 140.2 | 1.89 |
|  | 126.1 | 1.79 |
|  | 103.6 | 1.96 |

The concentration and A260/280 values of RNA samples was measured with NanoDrop One (Thermo Fisher Scientific, Waltham, MA, USA).
